# B cells and the stressed brain: emerging evidence of neuroimmune interactions in the context of psychosocial stress and major depression

**DOI:** 10.3389/fncel.2024.1360242

**Published:** 2024-04-08

**Authors:** Elizabeth Engler-Chiurazzi

**Affiliations:** Department of Neurosurgery and Neurology, Clinical Neuroscience Research Center, Tulane Brain Institute, Tulane University School of Medicine, New Orleans, LA, United States

**Keywords:** stress, major depression, immune system, B lymphocyte, brain

## Abstract

The immune system has emerged as a key regulator of central nervous system (CNS) function in health and in disease. Importantly, improved understanding of immune contributions to mood disorders has provided novel opportunities for the treatment of debilitating stress-related mental health conditions such as major depressive disorder (MDD). Yet, the impact to, and involvement of, B lymphocytes in the response to stress is not well-understood, leaving a fundamental gap in our knowledge underlying the immune theory of depression. Several emerging clinical and preclinical findings highlight pronounced consequences for B cells in stress and MDD and may indicate key roles for B cells in modulating mood. This review will describe the clinical and foundational observations implicating B cell-psychological stress interactions, discuss potential mechanisms by which B cells may impact brain function in the context of stress and mood disorders, describe research tools that support the investigation of their neurobiological impacts, and highlight remaining research questions. The goal here is for this discussion to illuminate both the scope and limitations of our current understanding regarding the role of B cells, stress, mood, and depression.

## 1 Introduction

As appreciation for the complexity of neuroimmune interactions continues to grow, there is increasing evidence that B lymphocytes may play important roles in central nervous system (CNS) structure and function across the lifespan (Dwyer et al., 2022). Indeed, pathogenic as well as protective effects of B cells have been reported in the context of neurological and spinal cord injuries (Engler-Chiurazzi et al., 2020; Plantone et al., 2022; Maheshwari et al., 2023; Malone et al., 2023), autoimmune diseases (Jain and Yong, 2022), and brain aging, neurodegeneration, and dementia (Plantone et al., 2022), to name just a few examples. Neuroinflammation is a shared feature of many of the neurological diseases in which B cells are implicated (Bersano et al., 2023). And yet, while systemic and neuroinflammation is being increasingly linked to the response to psychosocial stress and the manifestation of chronic stress-related conditions (Maes, 2011; Wohleb et al., 2016; Herkenham and Kigar, 2017; Dantzer, 2018), the role of the B cell in this context is not well understood.

This discussion will focus on B cells in the context of psychosocial stress and the impacts these cells may have on stress disorders, including major depression (MDD). The sections below will reiterate the clinical significance posed by stress-related disorders, review B cell functional roles within the immune system, describe clinical and foundational observations implicating B cell-psychological stress interactions, discuss potential mechanisms by which B cells may impact brain function in the context of stress and mood disorders, describe research tools that support the investigation of their neurobiological impacts, and highlight remaining research questions. The goal here is that this discussion will illuminate both the scope and limitations of our current understanding regarding the role of B cells, stress, mood, and depression.

## 2 Brief background of B cell development and activation

**Figure 1:**
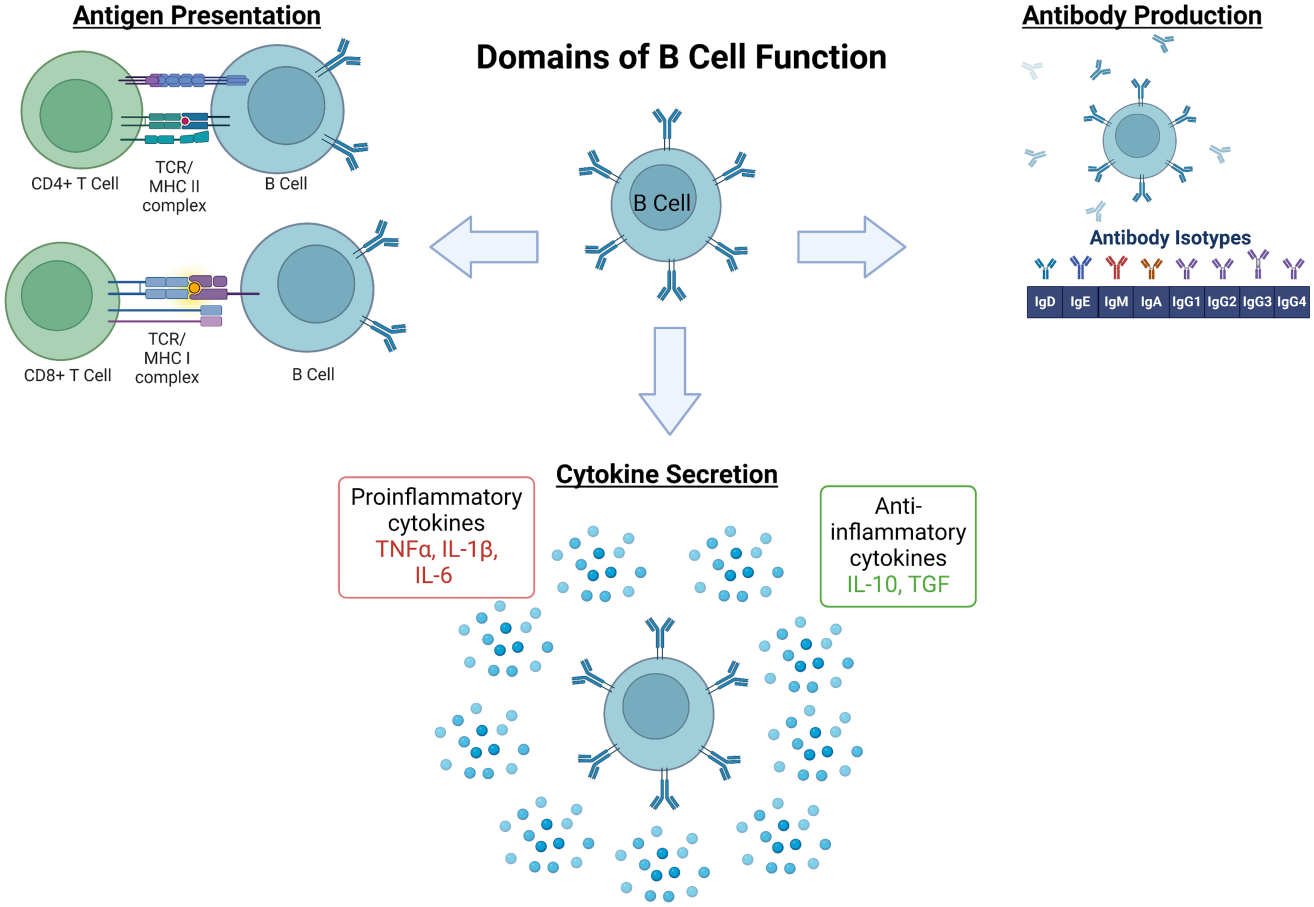
Domains of B lymphocyte function in the immune system. The process governing development, activation, and maturation of B lymphocytes is multifaceted and complex. Once activated by their cognate pathogenic signal, B cell subset functions within the broader immune response include (1) antigen presentation via major histocompatibility complexes (MHC) to effector immune cells, namely CD4^+^ or CD8^+^ T cells, (2) antibody production, and (3) pro- or anti-inflammatory cytokine secretion. These functions coordinate and potentially amplify, restrain, or resolve the broader immune response (Engler-Chiurazzi et al., 2020), and also serve as the foundation for immune memory to a given immunogen (Plantone et al., 2022). IL, Interleukin; TCR, T Cell Receptor; TGF, Transforming Growth Factor; TNF, Tumor Necrosis Factor. Figure created with BioRender.com.

The process underlying B cell development, activation, maturation, and effector functions is complex and has been extensively reviewed elsewhere (Meinl et al., 2006; Jain and Yong, 2022). In brief here, the majority of B cell development begins in the bone marrow. There, hematopoietic stem cells differentiate into pro- and then pre-B cells, proceeding through several key developmental stages of random recombination of the B cell receptor heavy and light chains to generate lymphocytes with responsivity to a huge diversity of immunogens. Self-reactivity, determined by at least three checkpoints (immature, transitional, and activated naïve B cell stages) must be lacking for survival and migration to secondary immune organs as naïve B cells. Naïve B1 B cells have limited B cell receptor diversity and are predominantly found in the peritoneal and pleural cavities. B2 follicular naïve B cells comprise the majority of B cells in lymphoid organs, namely the spleen. Following encounters with their antigen, a subset of activated B cells migrate to germinal centers (GCs) to undergo somatic hypermutation and class switch recombination to support affinity maturation. From there, maturing B cells become memory B cells or antibody secreting cells. A separate subset of antigen activated B cells will mature into short-lived plasmablasts outside of lymphoid structures while others become long-lived plasma cells (PC). Of note, following encounter with an antigen, activation can take place in T cell-dependent or -independent cascades depending on the B cell subset. Once activated, the functions of various B cell subsets can be classified into several domains: (1) producers of antibodies that, once created and bound to antigen, promote complement signaling as well as antigen neutralization, antigen opsonization (coating with antibodies), and ultimately destruction by other immune cells, (2) antigen presenters, and (3) cytokine and trophic factor secretors, (4) immune response regulators, and (5) memory cells that enable a more rapid and specific threat response in future encounters with that specific antigen (Figure 1). However, it is noteworthy that there is significant overlap and redundancy among the subset populations with regards to functional roles in response to immunogenic stimuli.

## 3 Clinical significance of stress-related disorders

Mood disorders, including MDD, are a significant global health issue. Lifetime prevalence of mood disorders has been reported to be nearly 10% (Steel et al., 2014; World Health Organization, 2017) and it was recently estimated that more than 320 million people currently are affected by MDD worldwide (Ferrari et al., 2013). Problematically, the burden of mood disorders appears to be increasing. Indeed, between 2005 and 2018 in the United States alone, the number of adults with MDD increased from 15.5 to 17.5 million patients; the annual costs associated with this condition (including direct, suicide-related, and workplace costs) are estimated at $326 billion (Greenberg et al., 2021), a nearly 38% increase from previous cost estimates (Greenberg et al., 2015). While treatment interventions including antidepressant drugs are available, there is significant heterogeneity with regards to latency to symptom amelioration and in their response efficacy profiles (Trivedi et al., 2006; Al-Harbi, 2012). These factors have coalesced to make MDD a leading cause of disability worldwide (Friedrich, 2017) and a significant source of human suffering. There is, therefore, an urgent need to improve understanding of its pathogenic mechanisms and develop novel, more effective treatment interventions.

## 4 Evidence supporting a role for the B cell in stress and depression

Although once considered “immune privileged,” a compelling body of literature indicates that the CNS and the peripheral immune systems engage in bidirectional communication, profoundly influencing one another during homeostasis and in pathological/diseased neurological states (Pavlov et al., 2018), including those associated with chronic stress and MDD. Indeed, it is now accepted that inflammatory cascades mediated by brain resident microglia as well as peripheral innate inflammatory and adaptive T cell-mediated arms of the immune system significantly contribute to MDD, at least in some patient subsets (Maes, 2011; Wohleb et al., 2016; Herkenham and Kigar, 2017; Dantzer, 2018). Is there evidence also implicating B cells?

### 4.1 Observations among clinical populations

Interest in interrogating immune underpinnings of mood began in the late-1980s/early 1990s, when initial pioneering studies revealed distinct patterns of peripheral immune cell profiles among patients with MDD and mentally healthy controls (Maes, 2011; Wohleb et al., 2016). Indeed, Maes et al. (1990) noted that MDD patients displayed elevated levels of cluster of differentiation (CD)-4^+^ T cells, higher circulating soluble interleukin (IL)-2 receptor levels, and higher percentages of cells expressing CD25 (IL-2 receptor). As well, elevated levels of circulating cytokines, principally tumor necrosis factor (TNF)-α, IL-1β and IL-6, have been consistently reported among some subsets of depressed populations (Dowlati et al., 2010; Köhler-Forsberg et al., 2017). Depression-associated profiles of some immune cell populations and proinflammatory cytokines also normalized among patients responsive to pharmacological therapeutic intervention (Roumestan et al., 2007; Arteaga-Henríquez et al., 2019). Antidepressant treatment with co-administration of anti-inflammatory agents, such as non-steroidal anti-inflammatory drugs, statins, or cytokine inhibitors, improved depressive symptoms and MDD remission rates (Köhler‐Forsberg et al., 2019). However, high levels of inflammatory biomarkers are also often associated with poor responsiveness to serotonin (5-HT) targeting interventions (Arteaga-Henríquez et al., 2019), possibly accounting for the large heterogeneity in antidepressant efficacy for symptom remission in this population (Trivedi et al., 2006; Al-Harbi, 2012; Akil et al., 2018). Finally, observations that autoimmune patients prescribed inflammatory interventions developed rapid and profound depressed mood provided strong empirical support for immune-MDD associations (Pryce and Fontana, 2017).

At least initially, available evidence suggested that B lymphocytes were not consistently impacted by MDD. For instance, studies have reported both an increase or a decrease in B lymphocyte counts among students experiencing exam stress (Maes et al., 1999; McGregor et al., 2008). Some studies found no difference in B cell counts between MDD patients and controls (Darko et al., 1988) while others reported increased total or subset B cell counts (Maes et al., 1992) and still others showed reduced numbers (Pavón et al., 2006). Later, Ahmetspahic et al. (2018) identified that while numbers of total B cells were not altered among MDD patients relative to healthy controls, there were reduced counts of IgD^+^CD27^−^ naïve, CD1d^+^CD5^+^ regulatory-like, and CD24^+^CD38^hi^ B cell populations. However, these early studies were limited by a number of factors, including a highly heterogenous patient population, small sample sizes, and a focus on circulating immune cell profiles identified with lineage markers and immune cell phenotyping tools available at the time. Interestingly, a recent meta-analysis revealed elevated B cell counts in MDD patients (Foley et al., 2023). These findings are summarized in Table 1.

**Table 1:** Summary of clinical data regarding B cell changes in stress and depression.

Domain	Study population	Impact to circulating B cells profiles	Citation
Psychological stress	University students (*N* = 38) weeks before, immediately prior to, and weeks after examination periods; classified as stress reactors or non-reactors based on Perceived Stress Scale scores	In stress reactive students, CD19^+^ B cells were increased relative to their baseline counts	Maes et al. (1999)
	Doctoral trainees 3 days following qualifying exams and matched community controls (*N* = 10/group)	Mean CD19^+^ lymphocyte percentage was reduced	McGregor et al. (2008)
MDD	Patients with MDD and age-, sex-, and race-matched controls (*N* = 11/group)	B cells not changed	Darko et al. (1988)
	Minor (*N* = 14), simple major (*N* = 12), and melancholic (*N* = 12) depressed patients (*N* = 38) and normal controls (*N* = 10)	Pan (CD19^+^) B cells increased in melancholic patients	Maes et al. (1992)
	MDD outpatients (*N* = 33) and non-depressed controls (*N* = 33) matched for age, ethnicity, and demographic characteristics	CD3^−^CD19^+^ B cells were reduced	Pavón et al. (2006)
	MDD patients (*N* = 37) before and 6 weeks after mood-modulating intervention (antidepressants, benzodiazepines, mood stabilizers, and/or antipsychotics) and healthy donors (*N* = 27)	Prior to treatment, frequencies of B cells (CD19^+^) were not altered compared to controls but IgD^+^CD27^−^ B cell proportions were reduced CD24^+^CD38^hi^ immature transitional B cells were reduced CD38^hi^IgD^+^ Bm2 B cells were reduced CD1d^+^CD5^+^ B cells were reduced CD5 expression increased on B cells in treatment responsive patients	Ahmetspahic et al. (2018)
	Depressed patients before (*N* = 31) and after (*N* = 10) 52 weeks of antidepressant treatment (various selective serotonin reuptake inhibitors), and healthy controls (*N* = 22)	No differences in B cells prior to treatment between patients and controls B cell counts increased with treatment relative to their own baseline values and those of healthy controls	Kolan et al. (2019)

### 4.2 Preclinical associations between B lymphocytes and the response to stress: focus on glucocorticoid signaling through the hypothalamic–pituitary–adrenal axis

#### 4.2.1 Shifts in B cell number and functional profiles with pharmacological and behavioral stress exposure

The response to psychological stress is generally considered to be mediated by engagement of the hormones involved in the hypothalamic–pituitary–adrenal axis (HPA; namely glucocorticoids), the sympathetic nervous system via catecholamines (discussed below), and more recently cytokines and immune cells. While glucocorticoids were historically considered immunosuppressive, collective evidence now indicates that stress-immune interactions, including those pertaining to B cells, are dynamic and complex (Kovacs, 2014). Indeed, like T cells, B cells of virtually all developmental stages express the cellular machinery [namely glucocorticoid (GR) and mineralocorticoid (MR) receptors] to respond to stress signals (Gruver-Yates et al., 2014) and studies have evaluated the effects of stress exposure on the number and function of B cells in key immune- and stress-related tissues. For instance, findings from several *in vitro* studies indicate that B cells, and particularly immature B cells, are susceptible to apoptotic consequences with exposure to high levels of corticosteroids (Garvy and Fraker, 1991; Lill-Elghanian et al., 2002; Igarashi et al., 2005). Further, in long-term cultured B lymphoblastoids collected from MDD patients or healthy controls, basal GR expression was elevated in depressed patients but showed a larger expression reduction when exposed hydrocortisone stimulation (Henning et al., 2005). *In vivo,* circulating B lymphocyte counts are reduced with administration of exogenous corticosterone (Dhabhar et al., 2012; Gruver-Yates et al., 2014) and tablet implant-based elevations in corticosterone levels in young adult male mice resulted in profound loss of bone marrow B cell subsets (Garvy and Fraker, 1991).

Behaviorally-induced glucocorticoid signaling has been shown to have a profound impact on B cells. For example, in adult male Sprague–Dawley rats, circulating B cell numbers initially increased in the minutes following a single acute restraint stress exposure; numbers then decreased over 2 h to below baseline levels, which the authors considered indicative of lymphocyte trafficking into tissues (Dhabhar et al., 2012). Stefanski and Engler (1998) noted reduced circulating B cell numbers with exposure to 2 h of social defeat stress in Long Evans rats, suggesting similar lymphocyte consequences in other stress conditions. B cell changes have also been reported in the context of chronic stress paradigms in a tissue-dependent manner. Although splenic and bone marrow B cell counts remained steady, 14 days of restraint for 2, 3, or 5 h/day significantly reduced B cell numbers in blood and thymus (Domínguez-Gerpe and Rey-Méndez, 2001). Leveraging a nine-week chronic variable stress paradigm, Gurfein and colleagues noted that stress exposure increased the frequency of immature and marginal zone, but decreased the frequency of follicular, B cells; interestingly total B cell counts were not altered (Gurfein et al., 2014, 2017). Notably, many of these effects are altered by environmental enrichment, an intervention known to attenuate the negative effects of stress paradigms (Fox et al., 2006). Corticotrophin releasing hormone (CRH)-transgenic mice, a well-established genetic model of stress in which persistent activation of the HPA axis is associated with increased corticosterone production and avoidance behaviors seen in chronically stressed organisms (Stenzel-Poore et al., 1992, 1994), display fewer total splenic, circulating, and bone marrow B cell counts (Murray et al., 2001). More specifically, B cell profiles tended to be skewed, with transgenic animals having higher proportions of immature cells within the tissues assayed. Finally, functional capacity of B cells may be affected by stress as chronic restraint also impaired GC responses including immunoglobulin (Ig)G1 antibody production, an effect which was prevented by GR blockade (Sun et al., 2019).

Appreciation for the importance of brain-spleen interactions in CNS health and disease is growing (Wei et al., 2022). Splenic changes associated with stress/inflammatory exposure include altered gross morphology, shifts in profiles of distinct immune cells, and changes in cytokine secretion patterns (Wei et al., 2022). For instance, repeated social defeat stress increases spleen weights, promotes hematopoietic stem cell progenitor release from bone marrow and recruitment to spleen, and induces splenomegaly with significant increases in CD11b+, natural killer, and granulocyte cell populations observed among stress-susceptible mice (McKim et al., 2018; Zhang et al., 2021). Similarly, systemic inflammation, like that induced by the bacterial infection mimic and well-established model of sickness behavior, lipopolysaccharide (LPS) (Dantzer, 2018), can profoundly alter splenic morphology and levels of circulating proinflammatory cytokines (Zhang et al., 2020). Stress exposure prior to LPS administration primes inflammatory responses in systemic and central macrophages (Johnson et al., 2002; Frank et al., 2007). It is noteworthy that stress and inflammation influences on splenic immune populations can vary by cell type and even cell subtype (Gurfein et al., 2014, 2017). Moreover, LPS is associated with inducing sickness and depressive-like behaviors and these behaviors appear to be mediated by activation of indoleamine 2,3-dioxygenase and other components of the kynurenine pathway (Bluthé et al., 1992; O'Connor et al., 2009; Remus and Dantzer, 2016).

Yet, while brain-spleen innervation patterns have been known for some time, and include some notable stress-related regions (e.g., locus coeruleus, central amygdala, and hypothalamic nuclei) (Cano et al., 2001), until recently very few studies have investigated the convergence of stress exposure, splenic B cells and the brain. In an elegant series of experiments, Zhang et al. (2020) found that CRH expressing neurons in the central amygdala and paraventricular nucleus (PVN) of the hypothalamus signal though the splenic nerve to impact B cell maturation. Activation of these neurons when mice were exposed to a chronic regimen of daily brief elevated platform stress increased numbers of splenic PCs, a finding that was later replicated by Lynall et al. (2021) using a chronic social defeat stress paradigm. PVN CRH-expressing neurons were also found to control acute restraint stress-induced shifts in peripheral lymphocyte pools (Poller et al., 2022). Indeed, stress exposure was associated with transiently reduced circulating splenic, and lymph node, B cells that homed to bone marrow. Excitingly, these findings demonstrate top-down brain control of peripheral adaptive immunity in response to certain types of psychosocial stress and lay mechanistic foundations for the continued exploration of these associations.

In addition to glucocorticoids, mineralocorticoids impact the stress response and MDD (de Kloet et al., 2016). Although aldosterone (a key MR ligand) has been reported to increase B cell activation and recruitment, the consequences of MR expression and binding on B cell function, especially in the context of stress, has been largely unexplored (Bene et al., 2014; Ferreira et al., 2021). Emerging data suggests that activation of MRs may play critical roles in modulating stress response resolution and promoting resilience (ter Heegde et al., 2015). Thus, future interrogation of the interplay between B cell and MR signaling will be key to discerning the full scope of stress-B cell interactions.

#### 4.2.2 Genetic modification of immune function alters stress responsivity

Further support for a role for lymphocytes in the response to stress comes from findings leveraging mouse models genetically modified to capture specific immune phenotypes. Studies in lymphocyte-deficient mice (nude, scid, or Rag −/− mice) have noted deficits in adaptability to stress; reconstitution of various lymphocyte populations in these mice generally implicated the absence of T cells in mediating these deficits (Cohen et al., 2006; Beurel et al., 2013; Rattazzi et al., 2013; Clark et al., 2014; Brachman et al., 2015). Importantly, (primarily T) lymphocytes from stress-exposed mice can modify the behavioral response to stress when adoptively transferred into lymphocyte deficient subjects (Brachman et al., 2015; Scheinert et al., 2016).

However, some evidence may support an association between the absence in B cells and pro-stress susceptible phenotype. Along with CD38, CD157, also known as bone marrow stromal cell antigen-1 (aka bone marrow stromal cell antigen-1), plays an important role in nicotinamide adenine dinucleotide metabolism and cell signaling cascades (Lopatina et al., 2020). CD157 is expressed on a number of cell types, including central and peripheral immune cells but also many nestin-positive neural cells (Higashida et al., 2017). Interestingly, CD157−/− mice displayed pronounced maladaptive responses to forced swim stress, leading authors to conclude that CD157 may play an important role in anxiety and social avoidance (Lopatina et al., 2014). Notably, B cell development in these mice is impaired as is the B cell-driven antibody response to infection (Itoh et al., 1998). This suggests that B cell deficiency may be at least partially involved with maladaptive stress responses and social interactions.

### 4.3 Evidence for B cell presence in CNS sites in stress and depression

The presence of peripheral immune cells in healthy brain parenchyma is rare (Korin et al., 2017) though robust immune cell recruitment to CNS can take place in times of neuroinflammatory challenge (Londoño and Mora, 2018), such as stress and MDD (Medina-Rodriguez et al., 2023). The neurobiology of stress and MDD is complex, and potentially injurious changes to brain structure and function have been reported when the stress response becomes dysregulated. Indeed, while these cascades have been extensively discussed elsewhere (McEwen, 2017; Godoy et al., 2018; McEwen and Karatsoreos, 2020), some stress-related consequences include reductions in brain volume (Koolschijn et al., 2009), region-specific changes in neuronal excitability leading to hypertrophy or atrophy (Chaudhury et al., 2015), tract-specific white matter disruptions and demyelination (Breit et al., 2023), blood–brain barrier (BBB) disruption and increased permeability (Medina-Rodriguez et al., 2023), and impaired hippocampal neurogenesis (Schoenfeld and Cameron, 2015). These highlight just a few examples of the numerous CNS impacts reported in both acute and chronic stress conditions among human and preclinical populations. A key question to address here is Do B cells accumulate in brain or brain-adjacent tissues during stress or depression, and if so, what role do they play? Do they promote chronic stress cascades, support resilience to the negative consequences of stress, or both?

Much of our understanding of B cell accumulation in CNS comes from neurological aging, injury or disease models such as stroke, or autoimmunity. Indeed, in multiple sclerosis (MS), gut origin IgA^+^ PCs that mediate mucosal immunity represent at least some of the B cell subsets that are recruited to CNS border sites and accumulate at lesions (Fitzpatrick et al., 2020; Pröbstel et al., 2020). Immunosenescence is associated with a profound shift in the B cell compartment and the emergence of innate-like age-associated B cells that accumulate in meninges (Brioschi et al., 2021). During stroke, B cells traffic to brain and accumulate both in the infarcted tissue and also at sites distal to stroke damage (Ortega et al., 2020). The functional significance of this accumulation is still being interrogated given that several reports note neuroprotective and neurorestorative actions of B cells (Ren et al., 2011; Ortega et al., 2020) while others suggest that autoreactive B cells may promote long-term poststroke cognitive deficits (Doyle et al., 2015).

There is limited but emerging support for brain-B cell interactions in the context of stress and MDD. For instance, in a few small cohort studies evaluating post-mortem brain tissues of schizophrenic, mood disorder, MDD, or bipolar disorder patients, a subset of subjects from each diagnosis condition displayed elevated brain lymphocyte profiles (Bogerts et al., 2017; Schlaaff et al., 2020). More specifically, in mood disorders, T and B cells were elevated relative to non-psychiatric patient controls. Interestingly, B cells appeared to accumulate in hippocampus/parahippocampal region, pre/peri/postcentral gyrus area, central white matter, and entorhinal and inferior temporal regions. Finally, B cells accumulated in hippocampi of mice exposed to a prolonged (but not acute) foot shock-induced learned helplessness or chronic restraint relative to non-stress exposed mice (Beurel et al., 2018). Surprisingly, chronic restraint stress did not result in similar T cell elevations, suggesting that stressor type may influence immune cell type recruitment patterns.

In addition to blood or organ-derived B cells, recent data identifying local CNS/CNS-adjacent originating B cells could suggest an additional mechanism by which B cells respond to neuroinflammatory states. Indeed, Brioschi et al. (2021) identified that B cells represent approximately 30% of CD45^+^ cells in the meninges. In addition, they identified a population of CNS antigen educated B cells that are derived from a bone marrow niche in the skull and reach meninges through vascular channels from the calvaria. Schafflick et al. further identified the dura as a site of progenitor B cell residence (Schafflick et al., 2021). Interestingly, while chronic learned helplessness increased hippocampal B cell numbers, splenic total B cell counts were not affected (Beurel et al., 2018). This observation, while considering that meningeal B cells were reduced in mice exposed to chronic social defeat (Lynall et al., 2021), may implicate meningeal or other local CNS-adjacent lymphocyte pools as the source of these parenchymal infiltrating cells.

In addition to trafficking to secondary lymphoid organs, leukocytes including B cells influence the local immune response by migrating to inflamed non-lymphoid tissues and forming ectopic or tertiary lymphoid structures (ELS) (Londoño and Mora, 2018; Harrer et al., 2022). ELSs in the CNS were first discovered in meninges close to inflammatory lesions in the context of MS (Negron et al., 2020), where the trafficking of B cells via key chemokine ligand-receptor interactions (discussed below) was shown to potentiate disease progression (Magliozzi et al., 2007). Additional studies have since identified B cell aggregating ELS in neuropsychiatric lupus (Stock et al., 2019), stroke (Doyle et al., 2015), and acute or chronic spinal cord damage (Cohen et al., 2021), to name a few examples. Importantly, research regarding development of local CNS ELS is in its infancy. Whether such structures form in response to the neuroinflammatory state produced by stress and MDD is not yet known though one study in a mouse model of lupus, a model in which depressive-like phenotypes have been well-established (Gao et al., 2009), found evidence for ELS in choroid plexus (Stock et al., 2019).

## 5 Mechanisms by which B cells migrate to, access, and remain at sites of inflammation

To address the role of B cells in the stressed brain, it is important to determine the extent to which B cell recruitment, trafficking, and survival signals are engaged during the experience of stressful life events, and to discern whether these signals become dysregulated in chronic stress.

### 5.1 B cell recruitment and trafficking signals

Central to an effective immune response to challenge is the tightly regulated movement of immune cells within lymphoid organs and to sites of inflammation. This process is governed by chemokines (Stein and Nombela-Arrieta, 2005; Chen et al., 2018). Several chemokines are implicated in the recruitment of B cells to key organs and sites, including C-X-C motif ligand (CXCL)12 (and cognate receptors CXCR4/7), CXCL13 (CXCR5), CC chemokine ligand (CCL)19 (CCR7), and CCL21 (CCR7) (Irani, 2016; Harrer et al., 2022; Jain and Yong, 2022). These chemokines can be homeostatic (e.g., CXCL13, CCL21), regulating cellular trafficking during immune surveillance or inflammation-induced (e.g., CXCL9, CXCL10). They can support development (e.g., CXCL12), and can modulate migration through primary (CXCL12) and secondary lymphoid organs (e.g., CXCL13, CCL19, CCL21). Some chemokines (CXCL12, CCL19, CCL21) are considered to have key brain homing functions. Importantly, some chemokines regulate the movement of several immune cell subtypes, including T cells (e.g., CCL19, CCL21, CXCL10).

B cell trafficking chemokines play diverse roles in the CNS. CXCL12, CCL19, and CCL21 are constitutively expressed in CNS at low levels namely by neurons, glial cells, and vascular endothelial cells (Li and Ransohoff, 2008; Lalor and Segal, 2010; Milenkovic et al., 2019) while these in addition to CXCL13 may be inducible in neural cell types in a disease state-dependent fashion (Krumbholz et al., 2007; Irani, 2016). Indeed, spinal cord neurons produce CXCL13 in a preclinical ligation model of neuropathic pain (Jiang et al., 2016) but not in viral encephalomyelitis where microglia show upregulated *Cxcl13* gene expression (Esen et al., 2014). Various CNS cell populations are responsive to chemokine signals but regional distribution of chemokine receptors is varied and suggests distinct functional roles for each ligand-receptor system (Turbic et al., 2011). For example, CXCR4, and CXCR7 to a lesser extent, is expressed on a wide variety of CNS cell types (such as astrocytes, microglia, and neurons and neural progenitor cells) in the hippocampus and subventricular zone. Thus, it is perhaps unsurprising that the CXCL12-CXCR4/7 system facilitates proliferation and migration of neural progenitor cells during development and adulthood (Schönemeier et al., 2008; Sánchez-Alcañiz et al., 2011). CXCL12 can also act directly on cells as it has been shown to induce GABAergic activity in diverse neuronal populations (Guyon, 2014). CXCR5 expression is not well represented regionally in CNS tissues though other findings suggest that this receptor is expressed by most CNS cell types (Turbic et al., 2011; Stuart et al., 2015). CXCL13 expression was upregulated in neurospheres exposed to inflammatory cytokine challenge (Turbic et al., 2011) and other cellular sources of CXCL13 produced during inflammatory conditions include microglia (Irani, 2016; Londoño and Mora, 2018) and astrocytes (Szpakowski et al., 2022). Several investigations regarding CXCL13 and the brain center on its contributions to autoimmunity, with converging findings suggests that CXCL13-mediated recruitment of B cells is associated with disease severity, progression, and poor prognosis (Londoño and Mora, 2018). CXCL13 neutralization in systemic lupus prone mice improved object recognition cognitive function and attenuated forced swim depressive-like behavior (Huang et al., 2021). However, in stroke, CXCL13 levels support trafficking of B cells to brain where they may mediate neuroprotective effects (Monson et al., 2014; Ortega et al., 2020). Increased CXCL13 and CXCR5 in anterior cingulate cortex facilitated conditioned place aversion in a rodent model of neuropathic pain, suggesting that these cascades, and possibly B cells themselves may benefit aversive learning and memory under physical stress conditions on the water maze (Wu et al., 2019). However, B cell deficiency did not impact memory performance (Wolf et al., 2009). CXCR5^−/−^ knockout mice displayed enhanced adult dentate gyrus neurogenesis and possible anxiolytic effects as indicated by increased movement in general and in the inner zone of the open field (Stuart et al., 2015). CCL21 may also impact adult neuronal differentiation given the high expression of CCR7 in the subventricular zone and on astrocytes, microglia, and neurons (Turbic et al., 2011; Noor and Wilson, 2012). This position adjacent to the CSF-filled lateral ventricle may also poise this chemokine-receptor system to engage in T and B immune cell recruitment. Indeed, CCL21 is expressed on the surface of choroid plexus epithelium and on endothelial venules only during inflammation and may mediate T or other CXCR7 expressing cell transit through brain-CSF and BBB routes (Alt et al., 2002; Kivisäkk et al., 2004).

Levels of chemokines have been investigated in the context of stress and MDD (Milenkovic et al., 2019). The majority of the published clinical studies reveal consistent circulating or CSF expression elevations in CCL2 and CXCL8 chemokines in depressed patients (Eyre et al., 2016). Of note, *in vitro* evidence suggests that these chemokines along with CCL20 may also support B cell trafficking and possible entry into CNS (Alter et al., 2003; Jain and Yong, 2022). Some clinical studies noted higher CXCL12 in depressed men and women in comparison with controls (Ogłodek et al., 2014; Romero-Sanchiz et al., 2020). Similarly, one study in rats exposed to prenatal stress (dam received gestational bright light exposure) revealed that CXCL12 was upregulated in the hippocampus and prefrontal cortex (Ślusarczyk et al., 2015) and microglial CXCR4 expression was reduced with prenatal stress (Ślusarczyk et al., 2015). Stress exposure may also modulate expression of recruitment signal receptors on B cells. Poller et al. (2022) demonstrated that bone marrow homing restraint stress-exposed B and T cells unregulated expression of CXCR4. Further, Lynall et al. (2021) noted that meningeal B cells from chronic social defeat exposed mice showed trends toward increased expression of CXCL13 genes. Other generalized immune cell recruitment signals may support B cell trafficking to CNS. Indeed, pathogen challenge increased brain chemokine expression and facilitated B cell recruitment in a CXCL9- and − 10-dependent manner (Lokensgard et al., 2016); these chemokines have been found to be elevated in patients with high levels of anxiety and MDD (Leighton et al., 2018). Finally, a thorough investigation of B cell recruitment signaling in CSF is still outstanding but may warrant further direct interrogation given that under normal conditions, levels of chemokines in CSF are low but increase manifold with inflammatory challenge (Pilz et al., 2020; DiSano et al., 2021).

### 5.2 Mechanisms modulating B cell access to brain

The mechanisms regulating immune cell migration across tissues, namely the BBB, are complex and has been extensively discussed elsewhere (Marchetti and Engelhardt, 2020; Jain and Yong, 2022). In brief here, several primary factors appear to regulate B cell movement across the BBB. First, activated leukocyte cell adhesion molecule (ALCAM) expressed on the surface of the activated proinflammatory B cells as well as on endothelial cells supports B cell diapedesis across the BBB (Michel et al., 2019). Leukocyte function-associated antigen-1 (LFA-1) on the surface of B cells binds its ligand intracellular adhesion molecule-1 (ICAM1). Very late antigen-4 (VLA4) expressed on activated B cells has been well studied in autoimmune diseases. Further, in autoimmune models, B cells appear to be recruited to CNS in an antigen-independent manner (Tesfagiorgis et al., 2017) and do not take up residence in sites of inflammation. L-selectin (aka CD62L) and vascular cell adhesion molecule-1 do not appear to play a robust role in B cell movement across membranes (Alter et al., 2003). While mechanisms regulating immune cell trafficking across BBB were extensively reviewed, whether B cells engaged these mechanisms in times of immune surveillance was not discussed (Marchetti and Engelhardt, 2020).

Studies on the effects of stress exposure, and stress-associated hormonal signaling, on leukocyte adhesion mechanisms have yielded interesting observations (Ince et al., 2018). Following 12 days of chronic unpredictable stress, RNAseq data in prefrontal cortex revealed increased gene expression of *Alcam* in non-neuronal cell clusters as well as several types of inhibitory and excitatory neuronal populations (Kwon et al., 2022). Fifteen minutes of forced public speaking resulted in elevated LFA-1 on mixed lymphocytes, no impact to ICAM1 density, and a decrease in L-selectin on T cells (Goebel and Mills, 2000). Similarly, VLA-4, CD44, and LFA-1 expression in T cells was altered by a single 16–18 h restraint stress exposure (Tarcic et al., 1995). With the exception of (Dhabhar et al., 2012), in which acute restraint stress reduced circulating B cell numbers and induced a biphasic decrease in L-selectin expression on B cells in the 2 h following stress cessation, the impact of stress exposure on adhesion mechanisms specifically in B cells were not measured in these studies. Interestingly, administration of norepinephrine (NE, aka noradrenaline), the receptors for which are expressed on B cells, decreased the number of L-selectin (CD62L) positive B cells but increased circulating B cells negative for this cell adhesion marker without impacting overall B cell L-selectin expression.

It is noteworthy that the BBB is not the only barrier impacted by stress. Several studies have found that intestinal barriers are leaky among patients with psychiatric disease (Wasiak and Gawlik-Kotelnicka, 2023). Immune cells are critically involved in the extent of intestinal barrier integrity. Indeed, B cells support intestinal homeostasis through production of IgA and IgM antibodies, immune regulatory functions, and cytokine secretion; whether they play a protective, pathogenic, or mixed role in intestinal inflammatory conditions is the subject of intense investigation (Zogorean and Wirtz, 2023). Gut microbiota composition is closely related to intestinal permeability status, and shifts in microbiome taxa composition have been reported in MDD (Cheung et al., 2019). As appreciation for gut-brain-immune interactions in the context of stress and MDD continues to increase, novel interventions such as psychobiotics or fecal microbiome transfers may prove beneficial (Trzeciak and Herbet, 2021).

### 5.3 B cell survival and retention in CNS or CNS-adjacent sites

If cells are recruited to sites of inflammation, such as the stressed brain, another question remains: is there sufficient support for B cells to survive and remain there to enact their cellular functions?

#### 5.3.1 BAFF and APRIL

Two principal B cell survival signals are well described in the immunology literature (Vincent et al., 2013). B cell activating factor (BAFF; aka B lymphocyte stimulator, TNFα APOL-related leukocyte expressed ligand, and CD257), is a cytokine belonging to the TNFα ligand family. It is expressed by a variety of immune cell types and acts to activate B cells as well as to induce their proliferation and differentiation. Receptors for BAFF include BAFF-R, BCMA, and TACI, in order of ligand affinity. BAFF-R is expressed on immature B cells, while TACI is principally expressed on innate like B1 cells. Inadequate BAFF fails to activate B cells to mature and go on to produce antibody or enact other effector functions; excessive BAFF levels limit B cell apoptosis and may cause overproduction of antibodies, potentially leading to autoimmunity.

A proliferation-inducing ligand (APRIL, aka TNFSF13) is another B cell survival signal (Vincent et al., 2013). APRIL acts as a co-stimulator for B and T cell proliferation and acts via TACI and BCMA receptors. APRIL also promotes IL-10 production and regulatory functions of B cells (Hua et al., 2016). Within CNS, APRIL is expressed by astrocytes in postmortem MS patient brains (Thangarajh et al., 2007) and facilitated axon growth of hippocampal pyramidal and midbrain and striatal dopaminergic neurons during embryonic development (Osório et al., 2014; McWilliams et al., 2017). Expression of BAFF/APRIL receptors in the brain is unclear (Marella et al., 2022) though a recently identified BAFF receptor Noggo-66 was found to be expressed on neurons and possibly glial cells, and may be a negative regulator of cell function (Zhang et al., 2009).

BAFF and APRIL may play a role in stress. Indeed, increased anxiety-like behaviors (open field, elevated plus maze, novelty suppressed feeding), evidence of neuroinflammation (reactive astrocytes, activated microglia), impaired hippocampal neurogenesis, and disrupted long-term potentiation were found in BAFF overexpressing transgenic mice, a common preclinical model of autoimmunity (Crupi et al., 2010). In humans, one small cohort study reported reduced circulating BAFF in MDD patients; levels were elevated with antidepressant intervention (Schmidt et al., 2019). However, in a larger study of 3,221 subjects, plasma levels of BAFF were not significantly different between healthy control subjects and patients with schizophrenia or MDD, though BAFF levels were elevated among bipolar patients (Engh et al., 2022). Plasma levels of APRIL were lower in psychosis patients compared to healthy controls and significantly correlated with higher psychotic symptom load, though other studies have not found APRIL levels to differ (Crupi et al., 2010; Schmidt et al., 2019).

While CNS levels of BAFF and APRIL have not been well studied in the stress exposed brain, Lynall et al. (2021) noted that meningeal B cells from chronic social defeat exposed mice were lower in number but showed trends toward increased expression of BAFF genes. As the stressed brain represents a state of neuroinflammation (Liu et al., 2017) and collective evidence suggests that the inflamed CNS provides a pro-survival microenvironment for leukocytes including B cells (Meinl et al., 2006), perspectives gleaned from other neuroinflammatory conditions can inform our understanding of B cell interactions with stress and MDD. For example, following experimental stroke, BAFF expression is elevated in microglia (Li et al., 2017) and CD11b^high^ B cells recruited to brain after injury are known to regulate microglia activation, increasing their phagocytic capacity (Korf et al., 2022). BAFF ligand-receptor interactions have been found to support neuronal survival and impart neuroprotection in an animal model of amyotrophic lateral sclerosis, an effect that may be independent of B cells (Tada et al., 2013). Astrocytes also express BAFF and promote B cell activation in response to viral infection (Lokensgard et al., 2016) or in MS (Krumbholz et al., 2005). Interestingly, in animals exposed to experimental autoimmune encephalomyelitis (animal model of MS) and in MS patients, disease attenuating anti-CD20 treatment increased BAFF levels in serum, CSF, and leptomeninges (Wang et al., 2024). Further, the beneficial effects for gray matter and microgliosis were reversed when anti-BAFF treatment was given in conjunction with B cell depletion, suggesting that BAFF plays a neuroprotective role in MS (Wang et al., 2024). Whether other CNS cell types produce BAFF and support B cell survival, especially in the context of psychosocial stress or MDD, is not known and represents an important question to be answered.

#### 5.3.2 Growth factors

Neurotrophins, such as brain derived neurotrophic factor (BDNF), neurotrophic factor 3 (NT3), and nerve growth factor (NGF), play important roles in development, survival, and function of a wide variety of CNS and immune cell types. Of relevance here, evidence suggests that B cells express neurotrophin receptors p75TR, TrkA, TrkB, and TrkC in a subtype-specific pattern (Hillis et al., 2016). Neurotrophins have complementary and sometimes redundant impacts to B cells. BDNF supports bone marrow B cell maturation as well as mature B cell survival (D'Onofrio et al., 2000), while NGF, and NT3 to a lesser extent, facilitate proliferation, survival, differentiation, antibody production, and CD40 T cell co-stimulatory expression (Hillis et al., 2016). Importantly, B lymphocytes can also secrete neurotrophins and this may have neuroprotective effects against inflammatory insults (Kerschensteiner et al., 1999).

A large body of evidence has implicated growth factors in mood disorders and/or the response to antidepressants (Zhang et al., 2015; Ceci et al., 2021). For instance, BDNF and TrkB levels are reduced in hippocampi of depressed patients (Zhang et al., 2015) and BDNF knock out mice display increased depressive-like behaviors and also have fewer B cells (Schuhmann et al., 2005). NGF, a potent survival factor for memory B cells (Hillis et al., 2016), is increased in inflamed tissues and has been shown to be elevated in blood in response to acute stress (Ceci et al., 2021). However, chronic stress and depressed mood conditions are often (though not always) associated with downregulation of NGF. Like other growth factors, NT3 is highly expressed in brain regions implicated in MDD, such as hippocampus, and is often elevated in MDD brains (Zhang et al., 2015). Known to regulate BDNF expression, one study reported reduced NT3 mRNA expression in peripheral white blood cells of MDD patients (Otsuki et al., 2008). While these associations are interesting, the paucity of work directly addressing the convergence of stress/depression, growth factors, and B cells leaves several remaining questions as to the extent to which these three factors interact to influence mood. Moreover, still more work is needed to address how B cells engage with other growth factors, including insulin-like growth factor, platelet derived growth factor, and glial derived growth factor, in these contexts.

#### 5.3.3 Interactions with T cells

Interactions with CD4^+^ T helper cells support survival and differentiation of B cells as well as GC formation, B cell isotype switching and somatic hypermutation, and formation of long-lived antibody-secreting PC (Aloulou and Fazilleau, 2019). The classic mechanism by which these interactions take place is via CD40 binding with CD40 ligand (CD40L) expressed by CD4 T and B cells (among other cell types) under inflammatory conditions. Importantly, a number of studies have identified important roles for CD4 T cells in CNS, namely in modulating neurological function in the healthy, stressed, injured, or diseased brain (Fletcher et al., 2010; Filiano et al., 2017; Herkenham and Kigar, 2017; Rayasam et al., 2018). In fact, one of the most consistently reported immune compartment alterations in depressed patient populations is an elevated circulating CD4^+^/CD8^+^ ratio (Maes, 2011; Herkenham and Kigar, 2017; Khedri et al., 2020). Further, preclinical studies in lymphocyte-deficient mice (nude, scid or Rag −/− mice) have noted deficits in adaptability to stress; reconstitution of various lymphocyte populations in these mice generally implicates the absence of T cells in mediating these deficits (Cohen et al., 2006; Beurel et al., 2013; Rattazzi et al., 2013; Clark et al., 2014; Brachman et al., 2015). A variety of T cell subsets have been found in brain parenchyma following acute and chronic stress, including CD4^+^ T cells (Beurel et al., 2013; Peng et al., 2022; Medina-Rodriguez et al., 2023). T cells also robustly respond to glutamatergic signaling (Ganor and Levite, 2014), a neurotransmitter system that is emerging as a key contributor to MDD and a principal target for novel, fast acting antidepressants (Wang et al., 2021).

There is evidence that co-stimulatory pathways involved with T-B cell interactions are impacted in stress and depression. Basal platelet CD40 expression is higher in patients with MDD (Neubauer et al., 2013). As well, circulating soluble CD40L levels were elevated in first episode unmedicated MDD patients and these levels were reduced when antidepressant interventions were administered (Leo et al., 2006; Myung et al., 2016), though another study reported reduced CD40L serum levels in MDD patients (Zhang et al., 2023). CD40 activation induces sickness behavior and depressive-like phenotypes in mice, as revealed by reduced saccharine preference and impaired fear learning (Cathomas et al., 2015). Thus, while T cells in the stressed brain may exert their own effector functions, their presence may serve to facilitate stress-responsive actions of B cells, or vice versa- B cells may facilitate the CNS effector functions of T cells.

One potential though controversial action of B cells is that they can regulate the development and activation of subsets of T cells via OX40-OX40 ligand or inducible T cell co-stimulator (ICOS)-ICOS ligand interactions (Petersone et al., 2018). While mice exposed to a single 12 h session of restraint did not show differences in splenic OX40 protein expression (Ahmad et al., 2015), hippocampal ICOS mRNA expression was elevated in mice exposed to footshock induced learned helplessness (Beurel et al., 2018). B cells may be a potential source of both OX40 or ICOS ligands (Petersone et al., 2018), yet there is little known as to how either ligands are impacted by stress or depression. Of note, though ICOS ligand is the exclusive binding partner for ICOS, it can be produced by most antigen presenting cells (Wikenheiser and Stumhofer, 2016) and some barrier cell types. Whether B cells are a significant source of ICOS ligand production in response to stress is currently unknown.

### 5.4 B cell-neurotransmitter interactions

#### 5.4.1 Serotonin

B cells express receptors for a wide variety of neurotransmitters and this may have important implications in stress and depression. The serotonergic (5HT) system has been historically implicated in chronic stress disorders and functioned as targets of antidepressant action, though that association is not without controversy (Jauhar et al., 2023). B cells express 5HT receptors 1A, 2A, 3 and 7 along with the 5HT transporter (SERT) (Herr et al., 2017). 5HT stimulation of B cells appears to induce proliferation, likely in a 5HT-1A receptor dependent mechanism (Iken et al., 1995) that requires inflammatory gene transcription (Abdouh et al., 2001). Some evidence in B cell and lymphoma lines suggests that SERT expression increases in activated B cells enabling them to take up 5HT (Meredith et al., 2005) and transport it, potentially long distances. However, B cell-derived lymphoma cells failed to proliferate with 5HT stimulation and showed increased apoptosis with 5HT uptake (Serafeim et al., 2002; Kolan et al., 2019). Interestingly, antidepressant intervention restores some B cell population numbers in MDD (Hernandez et al., 2010; Ahmetspahic et al., 2018). Lacking expression of tryptophan hydroxylase 1, B cells do not appear to be able to synthesize 5HT like T cells (Herr et al., 2017). MDD patients consistently show reduced expression of SERT on peripheral lymphocytes that can be ameliorated at least in part with administration of one of several classes of antidepressant intervention (Urbina et al., 1999; Peña et al., 2005). As 5HT can also be synthesized in both the brain and the periphery via distinct mechanisms, this should be considered when trying to discern the convergence of 5HT, B cells, and stress (Herr et al., 2017).

#### 5.4.2 Norepinephrine/noradrenaline

In addition to regulating the immune response via stress signaling through the HPA axis, the brain leverages the sympathetic nervous system innervation of primary and secondary immune organs principally through release of NE (Kin and Sanders, 2006; Herkenham and Kigar, 2017; Chan et al., 2023). Antigen-mediated immune activation resulted in increased NE release and turnover in lymphoid organs, an effect that was partially blocked when NE signaling from brain to periphery was inhibited (Kohm et al., 2000). Immune cells located in these organs express NE receptors, specifically β2 adrenergic receptors (Kerage et al., 2019); moreover, this receptor is expressed much more highly on B cells than T cells (Kin and Sanders, 2006). Lymphocytes express the NE transporter (Mata et al., 2005), and dopamine (DA)-β hydroxylase, a key enzyme governing the conversion of DA to NE, is also found in B cells (Papa et al., 2017). This suggests that B cells can take up and also synthesize NE *de novo*.

In the context of public speaking stress, which elevated circulating NE levels, there was a trend toward elevated peripheral B cell counts but also a tendency toward less IgM produced in response to mitogen stimulation *in vitro* when compared with B cells collected pre-stress (Matthews et al., 1995). Retrograde viral tracing revealed that several stress/reward-associated brain regions, such as hypothalamus, hippocampus, amygdala, ventral tegmental area, and locus coeruleus, innervate bone marrow, a site of NE release (Dénes et al., 2005). Similar regions were reflected in brain-spleen innervation patterns (Cano et al., 2001), further supporting an association between NE, B cell immunity, and stress. In MDD patients, while NE levels were not altered, lymphocyte NE transporter expression was reduced (Mata et al., 2005). It may be that stress effects on at least some immune cells are mediated by both corticosterone and catecholaminergic stimulation as noted that adrenalectomy or catecholamine receptor antagonism prevent at least some stress-associated circulating leukocyte shifts (Engler et al., 2004). Interestingly, epinephrine (adrenaline), but not NE administration, reduced total B lymphocytes in circulation, suggesting these cells were trafficked into tissues (Dhabhar et al., 2012).

#### 5.4.3 Dopamine

DA, a key mediator of reward circuits known to be dysregulated in MDD (Russo and Nestler, 2013), is now appreciated to have potent immunomodulatory actions (Vidal and Pacheco, 2020). Relative to other immune cell types, B cells displayed the most robust expression of DA receptors (DRDs); DRDs 2–5 were expressed on B cells, with DRD3 expressed at the highest levels (McKenna et al., 2002). Stimulation of unique DRDs can exert distinct and sometimes competing effects within the immune system. For example, B cell DRD1 stimulation by CD4^+^ T follicular cells, found to be potent secretors of DA, reorganizes expression of co-stimulator machinery, facilitates GC synapses, and promotes B cell maturation (Papa et al., 2017). In a small cohort study of patients with rheumatoid arthritis, B cell DRD expression was lower than that of healthy controls, B cell DRD2 stimulation is negatively correlated with disease activity (Wei et al., 2015), and DRD2 and − 3 expression levels were increased among patients treated for 3 months with an anti-rheumatic disease modifying drug (Wieber et al., 2022). However, in female rheumatoid arthritis patients, B cells were shown to express DRD1, the frequency of DRD1 was correlated with severity of disease, and stimulation of these cells increased expression of proinflammatory factors, suggesting sex-specific effects with regard to DA-B cell interactions in this disease. Further, the consequences of DAergic stimulation of B cells may be disease context-specific, as MOG-induced mouse models of MS promoted CNS infiltration of DRD3 expressing B cells with regulatory/immunosuppressive and antigen presenting phenotypes (Prado et al., 2021). Expression of the DA transporter is found on lymphocytes and mice in which it is lacking show exaggerated memory B cell responses (Gopinath et al., 2023). Further, DA transporter expression on resting lymphocytes was reduced in schizophrenic and bipolar psychosis patients (Marazziti et al., 2010). Chronic stress can induce hyperexcitability in certain DAergic neuronal populations, namely in stress- and mood-related brain areas in which DA expression is high (including hippocampus, paraventricular nucleus, and striatum) (Russo and Nestler, 2013). Whether these stress-related consequences in DAergic signaling impact the number and function of B cells that are present in or trafficked to these regions and what that means for stress susceptibility or resolution is worthy of further exploration. However, evidence suggests this potential as designer receptors activated only by designer drug-induced activation of the ventral tegmental area increased IgM^+^ B cells and potentiated IgM antibody secretion with *E. coli* immune challenge relative to mice who did not receive neuronal activation (Ben-Shaanan et al., 2016).

#### 5.4.4 Glutamate and GABA

Dysfunction in the glutamatergic system, representing the major excitatory neurotransmitter of the CNS, has emerged as a key component of MDD development and potential novel target for fast-acting depressive symptom amelioration (Sarawagi et al., 2021). Indeed postmortem assessments of MDD patient brains revealed reduced size of the glutamatergic neuronal layer VI (Cotter et al., 2002) as well as reduced expression of glutamatergic receptor machinery in prefrontal cortex (Feyissa et al., 2009). Similarly, in MDD, size and density of various GABAergic interneurons are reduced in this and other regions implicated including the anterior cingulate and amygdala (Rajkowska et al., 2007; Tripp et al., 2011; Guilloux et al., 2012). Imaging studies of a variety of modalities have confirmed disruption in these neurotransmitter systems (Sarawagi et al., 2021) and recent meta-analyses revealed decreased levels of glutamate+glutamine in the anterior cingulate and medial frontal cortex as well as decreased GABA in occipital and prefrontal cortex of MDD patients (Luykx et al., 2012; Romeo et al., 2018; Moriguchi et al., 2019).

Emerging evidence suggests that B cells can be impacted by glutamatergic receptor ligands. Indeed, human B cells expressed GRIK2, −3, −4, and − 5 kainate receptor genes and activation of these receptors resulted in increased proliferation and IgE release (Sturgill et al., 2011). B cells may also express metabotropic glutamate receptors and ligand binding may mediate apoptotic cascades (Ma et al., 2015). Finally, B cells may express functional N-methyl-D-aspartate receptors (NMDA; GluN2A and B) (Torres et al., 2021). The functional significance of which is not currently well studied though one recent report noted that non-competitive antagonists impaired B cell migration, proliferation and Ig production but increased numbers of IL-10 secreting B cells (Simma et al., 2014). Further interrogation of these complex interactions will be important considering the recent regulatory approvals for glutamatergic modulation in patients with refractory MDD (Sarawagi et al., 2021).

B cells may also interact with the GABA system, the key inhibitory neurotransmitter of the CNS (Sarawagi et al., 2021). Indeed, B cells can take up glutamine and process it to produce glutamate or GABA; CD8^+^ T cell or macrophage proinflammatory antitumor responses were dampened as a result (Zhang et al., 2021). Importantly, GABA deficiency has long been considered an important component of disordered mood (Sarawagi et al., 2021). Whether B cells in the CNS of behavioral stress-exposed or depressed organisms also secrete GABA and exert anti-inflammatory effects or neuromodulatory effects that regulate immune cascades present in stress/MDD-implicated brain regions is yet to be determined. For both glutamate and GABA, an informed understanding of B cell impacts will need to include the observation of B cell production of autoantibodies against these receptors must also be considered (Sun et al., 2020) (see below).

### 5.5 Potential CNS-directed effector functions of B cells

The next question to be answered is what might B cells be doing in the stressed CNS? The answer may be condition specific as B cells appear to be pathogenic drivers in autoimmunity and Alzheimer’s-like dementia whereas they may be neurorestorative in acute brain or spinal cord injury (Engler-Chiurazzi et al., 2020; Plantone et al., 2022; Maheshwari et al., 2023; Malone et al., 2023).

#### 5.5.1 B cell antibody production

A key effector function of B cells is their capacity to produce antibodies, including IgA, IgD, IgE, IgG, and IgM. Antibody production ability of B cells can have important functional consequences for CNS. For instance, B cells, and their production of IgM, may play a critical role in myelin development, a tightly regulated process dependent on local microenvironment signaling (Nishiyama et al., 2021). Tanabe and Yamashita (2018) identified that B cells, specifically B1a B cells, are recruited to choroid plexus and meningeal spaces via CXCL13 and supported oligodendrocyte proliferation via IgM secretion. Importantly, antibody titers may be altered by stress and in stress-related disease (Kronfol and House, 1989; Joyce et al., 1992; Song et al., 1994; Gold et al., 2012). Relative to mentally healthy control subjects, Gold et al. (2012) noted that depressed populations displayed reductions in serum IgA, but not IgM or IgG levels. However, Joyce et al. (1992) reported increased IgA in MDD patients. Exam and occupational stress was associated with elevated IgA, IgG, and IgM levels (Maes et al., 1999; Matos-Gomes et al., 2010). Methodological differences between sample populations and measurement approaches may account for some of the discrepancy between these studies, though recent meta-analyses confirmed that acute stress elevated Ig levels while chronically stressed humans showed age-related increased IgA or decreased IgM (Khan et al., 2021; Castro-Quintas et al., 2023). Mainly implicated in response to allergens, serum IgE levels are elevated in MDD patients (El-Ansary, 2022) and hydrocortisone increased IgE levels in IL-4 stimulated human lymphocytes (Wu et al., 1991). Though one study noted elevated IgD levels among MDD patients (El-Ansary, 2022), very little is known about how psychosocial stress or MDD impact IgD, likely due to its very low level of basal expression (Vladutiu, 2000). In mice, elevated platform stress-induced B cell profile shifts corresponded to an increase in IgG antibody titers in blood in the weeks following the stress exposure (Zhang et al., 2020). The functional consequences of these stress-induced shifts in Ig secretion for immunity are conflicting and may be Ig subtype specific. Shifted B cell profiles among CRH overexpressing transgenic mice were associated with less robust IgM and IgG antibody responses to a thymus-dependent, bacterial-like antigen immunization, yet there were no differences in survival when mice were exposed to gram-positive *S. pneumoniae* bacterial immune challenge (Murray et al., 2001). This suggests that stress-induced changes in B cell responsivity may not negatively impact overall immune response to at least some pathogenic challenges. Yet, speech making stress exposure increased the magnitude of allergen skin prick test wheal responses among allergic rhinitis (Kiecolt-Glaser et al., 2009).

Beyond classic immunological roles for immunoglobulins, clinical observations have noted that B cell-derived autoantibodies against CNS targets, namely glutamatergic but also GABAergic receptors, are implicated in several neurological disorders (Sun et al., 2020). For instance, NMDA receptor antibody encephalitis is associated with modest CNS perivascular and meningeal CD20^+^ B cells while CD138^+^ plasma cells have been observed in the CNS (Tüzün et al., 2009; Martinez-Hernandez et al., 2011), an effect supported by the observation of elevated CSF levels of CXCL13 observed in these patients (Leypoldt et al., 2015). Several reports note that patients with anti-NMDA encephalitis report poor psychosocial function (Blum et al., 2020; Nguyen et al., 2020) and humans (migrants) or mice with chronic life stress experience display serum NMDAR1-antibody (IgA) (Pan et al., 2021). As well, neuronal surface autoantibody expression has been implicated in a number of neuropsychiatric conditions, MDD included (Zong et al., 2017). Though the process governing immune tolerant B cell development is complex (Sun et al., 2020), two key sources of self-reactive autoantibodies have been identified: antibody-secreting plasmablasts from GCs in secondary lymphoid organs and bone marrow resident long-lived PCs (Hansen, 2022). This is important given the recent findings noting increased splenic PCs with acute and chronic stress in rodents (Zhang et al., 2020; Lynall et al., 2021), linking the possibility for B cells to play a significant role in stress exposure-induced CNS reactive autoantibody generation mechanisms. As to where these cells are located, the BBB disruption reported in stress and MDD provides a promising mechanism by which Igs (autoreactive or not), expressed at very low levels under normal conditions (Zong et al., 2017), are able to access the CNS. Intrathecal synthesis by PCs is another possibility that has yet to be fully discerned. It is worth noting that understanding in this field is still being developed and remains controversial (Vahabi et al., 2021), thus added clarity is likely to emerge with additional investigations in the coming years.

#### 5.5.2 B cell interactions with T cells

In addition to supporting activation and survival (above), B-T cell interactions take place within the context of antigen presentation via major histocompatibility complexes (MHC) (Chen and Jensen, 2008). Indeed, after internalization via pinocytosis or B cell receptor-mediated endocytosis, B cells display antigenic peptide via MHCs to the T cell receptor of their antigen-cognate T cell. This interaction serves discrete functions, dependent on MHC class. MHCI molecules are ubiquitously expressed on all nucleated cell types, including B cells (Rock et al., 2016) as well as neurons and glial cells in brain regions underlying mood and cognition (Elmer and McAllister, 2012). MHCI displays critical non-immune roles in cortical development, neurite outgrowth, synaptic regulation, and learning and memory (Shatz, 2009; Elmer and McAllister, 2012; Nelson et al., 2013). Further, MHCI expression on neurons is altered by cytokines, an important observation given that stress exposure induces proinflammatory shifts in cytokine profiles. When activated B cells present antigen via MHCI, CD8^+^ cytotoxic T cells are activated (Wieczorek et al., 2017) or in some cases tolerized (Bennett et al., 1998; Castiglioni et al., 2005). Like other professional antigen presenting cells, B cells constitutively express modest levels of MHCII surface molecules, though expression is substantially increased following cell activation or cytokine stimulation (Rock et al., 2016). Given the strong evidence for critical roles for T cells in MDD, namely the elevated ratio of CD4^+^/CD8^+^ T cells in MDD patients (Maes et al., 1990) and that T cell deficiency in mice increases maladaptive stress response behaviors which are ameliorated by T cell adoptive transfer (Cohen et al., 2006), antigen presentation and subsequent co-stimulation of T and B cells could have critical impacts on the stress response. Several genome wide association studies have revealed variants in expression of MHC-related genes among MDD patient populations (Woo et al., 2018; Wray et al., 2018; Li et al., 2019). Maes et al. (1992) also identified increased expression of MHC-II on the surface of T cells collected from blood of depressed patients. MHC expression has also been found altered in rodent models of stress. The percent of peritoneal macrophages displaying MHCII surface molecules was reduced in adult male mice exposed to a single session of 12–18 h of restraint stress (Zwilling et al., 1990). This effect appears to be mediated by glucocorticoid signaling as *in vitro*, MHCII expression in B cells exposed to corticosterone is reduced (Nugent et al., 2011). Whether similar changes are occurring on B cells in response to behavioral stress is not known.

#### 5.5.3 B cell cytokine production

B cells represent a heterogenous cell population with the capacity to secrete a variety of cytokines in response to immune stimulation and these secretion patterns can have fundamentally distinct consequences for immunity and for CNS (Hoffman et al., 2016; Jain and Yong, 2022). As elegantly depicted in 6, B cell-derived cytokines can promote macrophage activation (granulocyte-macrophage colony stimulating factor), support PC differentiation and survival (IL-6), regulate T cell differentiation, proliferation and inflammatory function (IL-6; transforming growth factor-β, and IL-35), facilitate formation of follicle-like structures (lymphotoxin-α) and modulate microglial cell activation (TNFα; interferon-γ). Indeed, proinflammatory B cells regulated microglia and macrophage proinflammatory cytokine expression in MS (Touil et al., 2018). In contrast, B regulatory cells have been recently been recognized to down-modulate inflammation, predominantly by restraining the inflammatory immune response via interleukin (IL)-10 secretion (Hoffman et al., 2016; Jain and Yong, 2022). This cytokine plays a protective role in various brain inflammatory conditions (Lobo-Silva et al., 2016), systemic levels are altered in MDD, and converging data support its critical role in mediating stress susceptibility (Mesquita et al., 2008; Gazal et al., 2015; Laumet et al., 2018). Importantly, in MS IL10-expressing B cells upregulated quiescence associated molecules (Touil et al., 2018). Such cell-to-cell interactions may exert important functional consequences considering the complex roles played by microglia in regulated CNS function under normal and MDD conditions (Wang et al., 2022).

## 6 Research tools

The pioneering insights gleaned from B cell interactions with CNS in other neurological conditions coupled with the availability of numerous B cell-modulating immunology research tools (Lee et al., 2021; Utset et al., 2021) poises the neuroscience field to rigorously interrogate mechanisms by which these cells may impact the response to stress. For instance, recent advances in spectral flow cytometry allow for more robust and comprehensive phenotyping of immune and resident cell panels in distinct tissues (Nolan, 2022). Administration of fluorescently labeled immune cells permits tracking of immune cell migration with a high degree of spatial specificity. Adoptive transfer techniques can be used to reconstitute transgenic animals lacking in distinct leukocyte populations (e.g., B cells absent in μMT) with distinct immune cell populations (e.g., B regulatory cells) or with cells which lack certain functional capabilities (e.g., IL-6 vs. IL-10 secreting capacity). Further monoclonal antibody (mAb)-mediated strategies to deplete distinct B cell populations, cytokines, or survival signals *in vivo* can differentiate functional impacts during adulthood from those during some critical developmental window. Some of the many available approaches including anti-CD19 or -CD20 mAbs (e.g., Inebilizumab; Rituximab; Ocrelizumab) are in trials or approved for use in autoimmune SLE or MS (Zhang et al., 2023). Moreover, various BAFF B cell survival signal inhibitors (e.g., Belimumab) are being developed or are currently deployed in clinical populations. Treatments developed for other therapeutic applications that are directed against other central [e.g., microglial depletion with colony stimulating factor 1 receptor inhibitors (PLX3397) (Basilico et al., 2022)] or systemic [e.g., anti-CD3 muromonab (Kuhn and Weiner, 2016)] immune cell populations can be used for discerning the extent to which interactions with other cells types is an important mechanism by which B cells influence the response to stress. MAbs directed against distinct cytokines are also available. A thorough understanding of the strengths and weaknesses of each approach is necessary to properly interpret results using these various research tools. For example, μMT mice exhibit reduced T cell numbers and functionality (Homann et al., 1998; Bergmann et al., 2001). Anti-CD20 B cell depletion (Rituximab) spares T cells (Uchida et al., 2004), innate-like T cell–independent B1 cells (Hamaguchi et al., 2005) and plasma cells (Hauser et al., 2008) while BAFF receptor targeting with TACI-IgG (Atacicept) robustly depletes the plasma cell compartment (Kappos et al., 2014). As appreciation for the role these cells play in orchestrating immune and neurological function during homeostasis and stress grows, this understanding should better inform our current and future interventions to support mental health and wellbeing.

## 7 Conclusion

**Figure 2:**
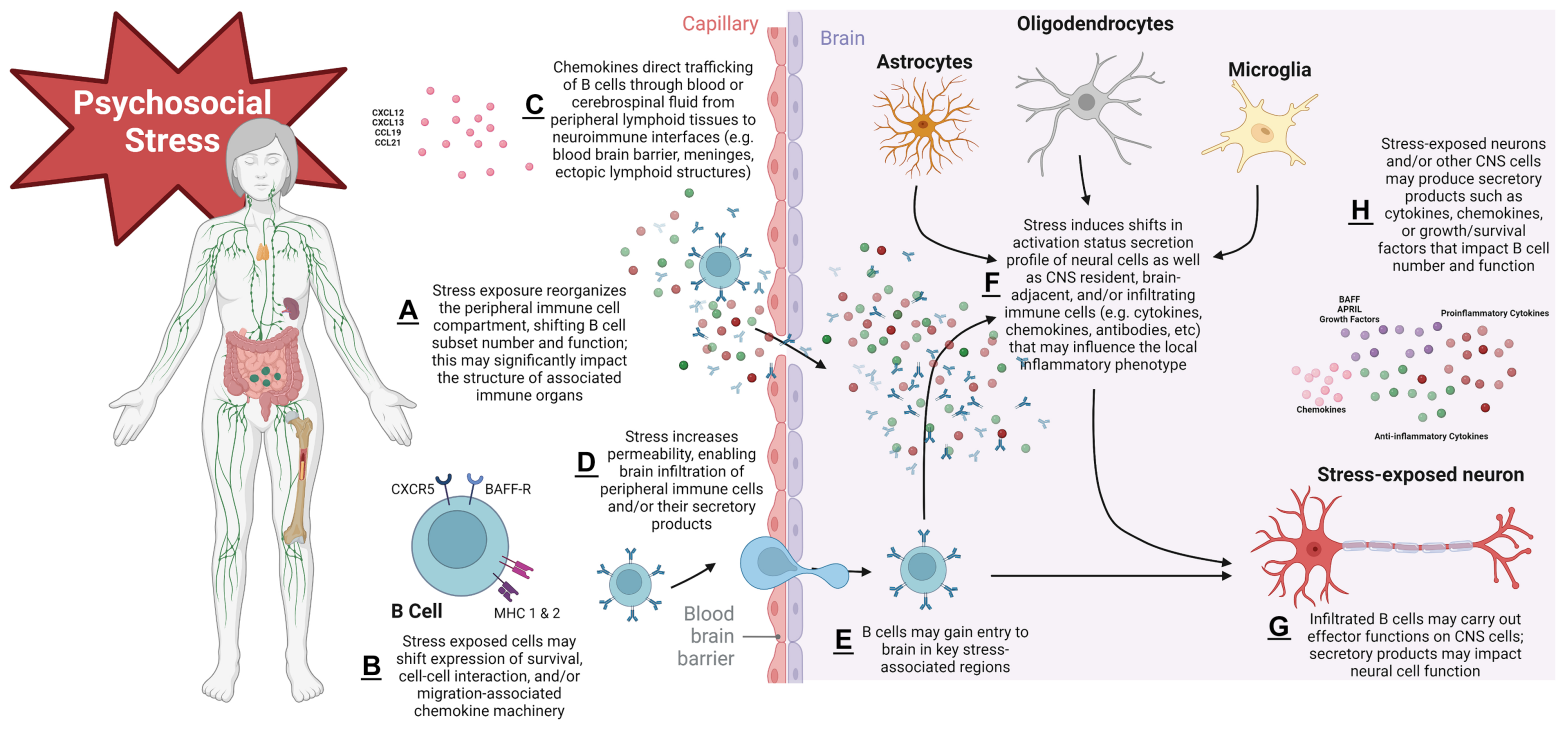
Proposed schema of mechanisms governing B lymphocyte impacts to the psychosocial stressed brain. Stress exposure initiates shifts in secondary lymphoid organs that reorganize the peripheral B cell compartment and alter number and function in a subtype-specific manner. This can have important morphological consequences for immune organs. Stress modifies expression of cellular surface machinery components that impact B cell development/survival, cell–cell interaction capacity, and migration. B cell trafficking signals govern the movement of cells to sites of inflammation in target organs, in this case the CNS. Stress exposure may render CNS protective barriers leaky, permitting infiltration of B cell secretory products (such as pro- and anti-inflammatory cytokines or antibodies) or cells themselves into brain parenchyma, where activation states and functional phenotypes of local resident CNS and/or immune cells have been altered. Cell survival signals may modulate the lifespan with which infiltrated cells have to carry out their effector functions on the stressed CNS. With repeated stress exposure, this cascade becomes dysregulated, leading to chronic stress disorders such as MDD. Figure created with BioRender.com.

Taken together, evidence above supports complex B cell-stress-brain interactions (Figure 2). Psychosocial stress exerts robust effects on B cells; these effects can vary on a number of factors including the nature of the stressor, the tissue, and the B cell subtype being assayed. Brain control of peripheral immune cell compartments suggest complex regulatory cascades are engaged to control this response. Perturbation of B cell systems may be associated with alterations to hedonic state that could render an organism susceptible to a depressive-like phenotype. Multifaceted signaling and trafficking systems are engaged with stress exposure, some of which may result in B cell migration through blood or CSF to CNS target sites such as meningeal ELS, circumventricular organs, or even parenchyma itself. Finally, given their ability to engage with key neurotransmitter systems, B cells may impact the stressed brain in functionally meaningful ways that will continue to be revealed as research in this area progresses in the coming years.

## Author contributions

EE-C: Conceptualization, Funding acquisition, Writing – original draft, Writing – review & editing.

## Figures and Tables

**Table:** 

5-HT	serotonin
ALCAM	activated leukocyte cell adhesion molecule
APRIL	a proliferation-inducing ligand
BAFF	B cell activating factor
BBB	blood–brain barrier
BDNF	brain derived neurotrophic factor
BMCA	B cell maturation antigen
CCL	CC chemokine ligand
CD	cluster of differentiation
CD40L	CD40 ligand
CNS	central nervous system
CRH	corticotrophin releasing hormone
CSF	cerebrospinal fluid
CXCL	C-X-C motif ligand
DA	dopamine
DRD	dopamine receptor
ELS	ectopic lymphoid structures
GABA	gamma aminobutyric acid
GC	germinal center
GR	glucocorticoid receptor
HPA	hypothalamic pituitary axis
ICAM	intracellular adhesion molecule
ICOS	inducible T cell co-stimulator
Ig	immunoglobulin
IL	interleukin
LFA	lymphocyte function associated antigen
LPS	lipopolysaccharide
mAb	monoclonal antibody
MDD	major depressive disorder
MHC	major histocompatibility complex
NE	norepinephrine
NGF	nerve growth factor
MR	mineralcorticoid receptor
MS	multiple sclerosis
NMDA	N-methyl-D-aspartate
NT3	neurotrophic factor 3
PC	plasma cell
PVN	paraventricular nucleus
SERT	serotonin transporter
TACI	transmembrane activator and calcium-modulator and cyclophilin ligand interactor
TNF	tumor necrosis factor
VLA	very late antigen
